# Spectrally Resolved Dynamics of Delayed Luminescence in Dense Scattering Media

**DOI:** 10.3390/ma18133194

**Published:** 2025-07-06

**Authors:** Mahshid Zoghi, Ernesto Jimenez-Villar, Aristide Dogariu

**Affiliations:** CREOL, The College of Optics & Photonics, University of Central Florida, 4304 Scorpius Street, Orlando, FL 32816, USA; mahshid.zoghi@ucf.edu (M.Z.); ernesto.jimenezvillar@ucf.edu (E.J.-V.)

**Keywords:** delayed luminescence, TiO_2_ nanoparticle, scattering media

## Abstract

Highly scattering media have garnered significant interest in recent years, ranging from potential applications in solar cells, photocatalysis, and other novel photonic devices to research on fundamental topics such as topological photonics, enhanced light–matter coupling and light confinement. Here, we report measurements of spectrally and time-resolved delayed luminescence (DL) in highly scattering rutile TiO_2_ films. The complex emission kinetics manifests in the non-exponential decay of photon density and the temporal evolution of the spectral composition. We found that while the energy levels of TiO_2_ nanoparticles broadly set the spectral regions of excitation and emission, our results demonstrate that the DL intensity and duration are strongly influenced by the inherent multiple elastic and inelastic processes determined by the mesoscale inhomogeneous structure of random media. We show that the lifetime of DL increases up to 6 s for the largest redshift detected, which is associated with multiple reabsorption processes. We outline a simple model for spectrally resolved DL emission from dense scattering media that can guide the design and characterization of composite materials with specific spectral and temporal properties.

## 1. Introduction

**Figure 1:**
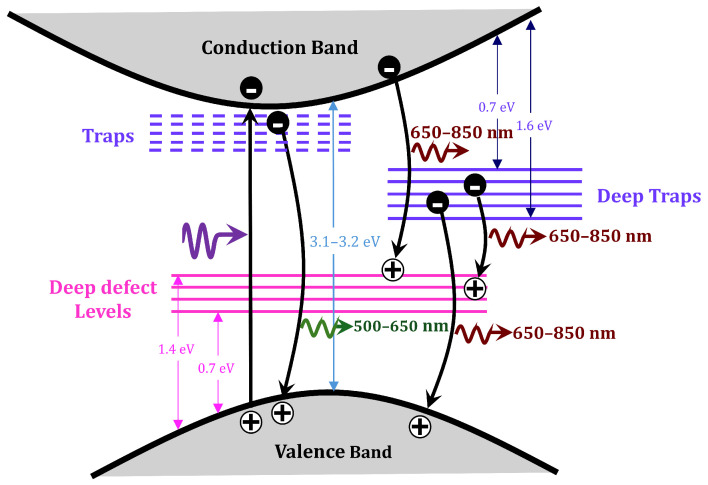
Electronic energy transitions leading to photoluminescence in TiO_2_ rutile [5,8].

Highly scattering media are commonly exploited in solar cells, photocatalysis, and a range of photonic devices aimed at enhancing light–matter coupling and light confinement. Titanium dioxide, in both rutile and anatase phases, is being widely used for its scattering properties in the visible and near-IR spectrum [1,2]. TiO_2_ has a large refractive index (2.8 to 2.5) and broad band gap (3.01 to 3.2 eV). When the energy of absorbed photons is higher than the band gap, electrons (*e*-) in TiO_2_ are transferred from the valence band (VB) to the conduction band (CB), generating electron–hole (*h*+) pairs. The *e*- and *h*+ recombination leads to luminescence bands in the visible and near-IR spectra, which have been ascribed to optical transitions with the participation of free carriers and impurity–defect states, self-trapped excitons [3], radiative recombination at chemical and structural defect states, or from donor–acceptor pairs [4,5]. A simplified schematic is shown in Figure 1. The photoluminescence (PL) spectrum and intensity in TiO_2_ have been shown to be strongly sensitive to the morphology of the TiO_2_ surface and chemical environment [5,6]. Despite extensive studies, the detailed photophysical mechanisms in TiO_2_ remain under debate [7].

The green–red PL (500–650 nm) has been attributed to oxygen vacancies on the surface and subsurface of TiO_2_ nanostructures [5]. The near-IR PL (650–850 nm) is ascribed to radiative recombination of mobile electrons in the conduction band or shallow bulk traps with immobile holes trapped in deep defect states (0.7–1.4 eV above the VB edge) or radiative recombination of electrons in deep traps (0.7–1.6 eV below the CB edge) with mobile holes in the VB [5]. Two possible mechanisms have been proposed for rutile near-IR photoluminescence that involve trapped electrons occupying mid-gap states positioned below the Fermi level or self-trapped holes located at oxygen atoms [5]. Near-IR (1.57–1.4 eV) time-resolved low-temperature (5 K) PL was studied for crystalline TiO_2_ powders, showing the decay rise time that can be fitted with two exponentials, one shorter $\tau_{1}\approx 60\;\mu s$ and other longer $\tau_{1}\approx 430\;\mu s$ [8].

The time scale of recombination processes in TiO_2_ is very sensitive to the excitation energy [9,10]. When excitation energy is below the band gap, electrons can be transferred from the valence band to shallow bulk traps or deep donor levels, resulting in localized electrons. Additionally, PL intensity, immediately after excitation, decays exponentially with a time constant of around 1–2 μs. For times longer than 10 μs, the intensity decay follows a power law [10]. This behavior has been explained by the contribution of trapped electrons, which remain stable even at room temperature, to the NIR luminescence [4]. That is, the photogenerated carriers are first captured by some stable traps, then relax slowly to the luminescent traps via trap-to-trap hopping, thereby contributing to the power-law decay processes.

The recombination rate strongly depends on the medium in which the TiO_2_ particle is embedded and can vary by 13 orders of magnitude [11,12] due to relaxation phenomena in TiO_2_ (charge–carrier recombination). In accordance with the literature [13], the following charge transfer steps have been proposed:

$${\mathrm{TiO}}_{2}+h\nu \to e_{CB}^{-}+h_{VB}^{+}$$

$$e_{CB}^{-}+{Ti}^{4+}\to {Ti}^{3+}\;\mathrm{trapped}\;\mathrm{electron}$$

$$h_{VB}^{+}+O^{2-}\to O^{-}\;\mathrm{trapped}\;\mathrm{hole}$$

On the other hand, in thick layers of TiO_2_ particles, multiple scattering events can affect this PL scenario due to a significant increase in the photon path lengths through the medium. Nevertheless, there are no systematic studies to date to address the consequence of strong multiple scattering on the temporal behavior of photon emission. In this paper, we provide direct experimental evidence of prolonged luminescence emission depending on the level of scattering.

Higher TiO_2_ particle concentrations generally increase scattering strength, defined as the inverse of the scattering mean free path *l*_s_, leading to longer photon path lengths and enhanced effective absorption and emission reabsorption [14,15]. The latter leads to PL redshift [16,17] and can also give rise to a delayed luminescence (DL) mechanism [18], which, unlike prompt photoluminescence, involves more intricate recombination dynamics and results in significantly longer-lived emissions governed by complex trapping, reabsorption, and scattering processes. A large overlap between the absorption and the PL spectra (i.e., small Stokes shift) facilitates the emission-reabsorption processes, which is expected at the tail of the TiO_2_ emission spectrum (700–850 nm). Therefore, when increasing scattering strength in a composite medium, one expects stronger reabsorption processes and a prolonged DL, which would be the consequence of longer photon path lengths.

Based on the proposed mechanisms, we conducted a detailed analysis of the experimental results. Specifically, we examined the PL decay rate of rutile TiO_2_ particles in scattering media with two different concentrations using a highly sensitive custom-built DL measurement setup. We employed two excitation sources with distinct pump wavelengths: 385 nm (above the bandgap) and 455 nm (slightly below the bandgap). PL emission was collected in transmission mode using spectral filters centered at 450, 500, …, 750, and 800 nm. Delayed luminescence with decay times ranging from milliseconds to seconds was measured. Using this setup, we investigated the spectrally resolved PL intensity and decay rates of samples. Although time-resolved photoluminescence investigations of TiO_2_ are available, they typically concentrate on ultrafast dynamics within picosecond to microsecond regimes [19]. Studies specifically examining spectrally resolved DL in TiO_2_ at extended timescales (milliseconds to seconds) are rare, making this work a potentially unique analysis [20].

Here, our main goal is to present experimental evidence of an unusually slow decay of emissions in highly scattering media and to phenomenologically interpret the fact that the DL intensity and the decay rates are sensitive to both elastic and inelastic light transport. We will show that a prolonged emission (slower DL decay rate) is observed as scattering strength increases, which can be associated with the enhancement of reabsorption from increased photon path lengths. A simple model that considers both the rutile energy levels described above and the scattering properties of TiO_2_ provides a comprehensive description of the main experimental results.

## 2. Systematic DL Measurements

Typical photoluminescence measurements involve rapid excitation and emission cycles. In large-scale, inhomogeneous materials, this limits the occurrence of steady-state cooperative effects such as sub-radiant directional memory in cooperative scattering [21]. Conversely, delayed luminescence involves prolonged emission lifetimes, allowing for the manifestation of cooperative interactions in disordered media, where light scattering leads to interference patterns that significantly influence emission properties. To investigate these phenomena, our custom-built measurement setup permits delayed-luminescence measurements on the scale of milliseconds and longer. This setup was specifically designed to overcome the limitations of standard photoluminescence spectrometers in detecting ultra-weak, long-lived emissions. By minimizing optical paths, temporally separating excitation and detection events, and employing a large collection aperture, we enhance sensitivity and enable DL decay profiling in scattering media. The system includes control over the excitation timing and emission data acquisition, ensuring that samples, regardless of their optical thickness, can achieve steady-state excitation, thereby enabling precise examination of these cooperative effects.

**Figure 2:**
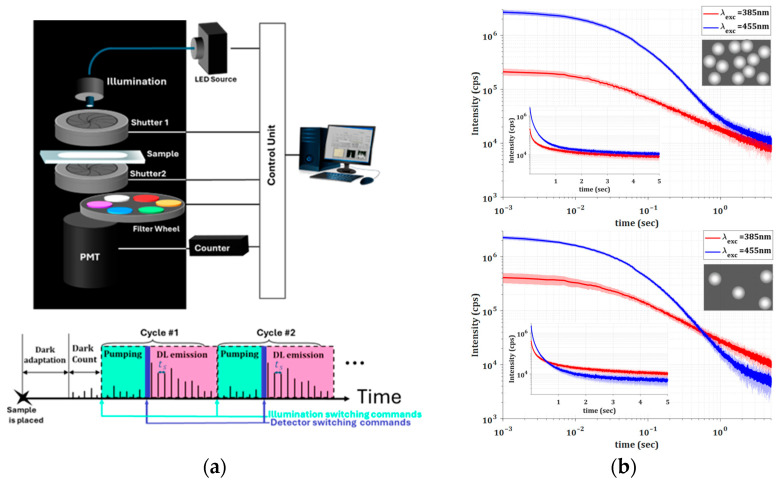
(**a**) Schematic of DL measurement setup and timing diagram. (**b**) Time-resolved total emission for Mediums 1 and 2, under different excitation conditions as indicated. The solid blue/red curves represent an average over 30 different measurements, while the shaded areas indicate the standard deviation across different realizations. Semi-logarithmic insets are included to highlight the non-exponential decay behavior. The inset cartoons illustrate schematically the differences in the density of scatterers between the two mediums.

The measurement setup is schematically depicted in Figure 2 and includes a multi-wavelength LED source for excitation (WFC-H4-0625-0530-0455-0385-000) (MIGHTEX, Pleasanton, CA, USA), two mechanical shutters with drivers for precise timing control (millisecond accuracy), and a filter wheel for achieving spectral resolution including 40 nm BW filters. Detection is performed using a high-bandwidth, low-dark-count photomultiplier tube (H11870-03) (Hamamatsu Photonics, Shizuoka, Japan) coupled to a high-rate counter, all enclosed within a dark chamber to practically eliminate any background emission. A customized computer code enables automatic remote operation of the entire system, ensuring ultra-low-dark-count measurements. In addition, the optical path lengths are specially designed to maximize the photon collection efficiency and, therefore, optimize the detection of emitted photons. The timing diagram, sketched in Figure 2, includes adjustable rates and delays. In all the reported time-resolved data, t = 0 corresponds to approximately 20 ms after shutting off the excitation light, accounting for the delay between closing the excitation shutter and opening the detection path. Due to millisecond-level variability in shutter performance, slight offsets existed between cycles. To correct this, all realizations were synchronized based on the maximum early detected emission, ensuring consistent timing across measurements. Time scales are reported in seconds.

The media examined were fabricated as follows. Suspensions of rutile TiO_2_ nanoparticles (~300 nm) were used in a water–polymer matrix, 60:40 in volume; the polymer consists of a 50:50 weight ratio of butyl acrylate to methyl methacrylate copolymers, supplied as a commercially available aqueous dispersion. The suspension, which is highly turbid in appearance, was stirred manually to maintain uniformity and prevent sedimentation prior to deposition. The mixture was applied onto BK7 cover glass slides using the doctor blade technique, with the blade spacing adjusted to produce films of approximately 375 µm thickness before drying. The films were then left to dry at room temperature for 24 h. Two samples were prepared with TiO_2_ weight concentrations of 12% and 4%, denoted as Medium 1 and Medium 2, respectively. For each sample, transmitted light from the 385 nm and 455 nm excitation sources was measured by spectrally filtering the radiation reaching the detector. These transmission measurements were conducted under continuous illumination, and the results are summarized in Table 1. The value of the transmission coefficient is determined by the ratio of the transmitted intensity to the incident intensity, being equivalent to the extinction coefficient.

For both media, the transmission increases significantly (approximately 28 times) for excitation at 455 nm compared to that at 385 nm. This is consistent with the rutile bandgap of ~3.02 eV (410 nm). Moreover, for both excitation wavelengths (385 and 455 nm), the higher concentration of TiO_2_ particles in Medium 1 leads to an approximately three times lower transmittance, which is the result of an increase in both elastic and inelastic scattering events. It is important to emphasize that the transmittance of the bare polymer matrix is considerably higher than that of the TiO_2_ particle layers due to the strong light scattering caused by the TiO_2_ particles. Consequently, the polymer matrix has a negligible effect on the DL signal. Direct measurements confirmed that the polymer matrix alone exhibited no delayed luminescence above the millisecond time scale within our detection limits.

During the DL measurement procedure, the samples were subjected to 30 cycles, each consisting of 5 s excitation followed by emission recorded for 5 s with sampling time $\tau_{s}=1\;ms$. The LED excitation source used had bandwidths of approximately 10 nm for 385 nm and 15 nm for 455 nm and excitation powers of 13.5 and 16 mW, respectively. Typical total emission results for the two different excitation wavelengths are shown in Figure 2b. As can be seen, DL responses are characterized by relatively long emission lifetimes and rather low numbers of emitted photons. The extremely long lifetimes (milliseconds to seconds) impose stringent limitations on the minimum detectable number of photons. Cyclical measurements and averaging techniques are therefore necessary to improve the signal-to-noise ratio.

Also notable in Figure 2b is the fact that the excitation wavelength matters, and, moreover, the emissions do not decay exponentially, which may indicate multiple possible emission mechanisms. The difference in the DL emissions measured for different excitation wavelengths are due to the rutile bandgap energy, which influence the probabilities of various electronic transitions, as previously discussed (see Figure 1). In addition, the fact that the overall DL intensities and the corresponding decay rates depend on the concentrations of TiO_2_ particles indicates that the scattering strength and the effective absorption significantly influence the propagation of both excitation and PL emission.

**Figure 3:**
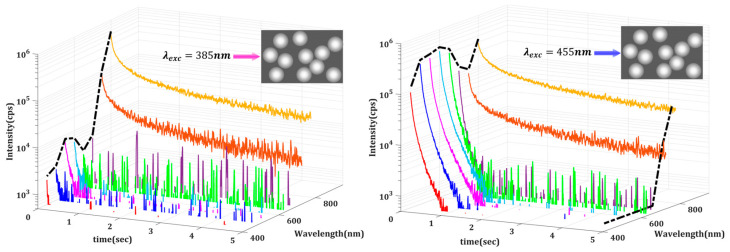
Corrected DL spectra for Medium 1 (12% TiO_2_ under two excitation wavelengths (385 nm, 455 nm), illustrating the wavelength-dependent decay dynamics. The emission was filtered at 450 nm (red), 500 nm (blue), 550 nm (magenta), 600 nm (cyan), 650 nm (green), 700 nm (purple), 750 nm (orange), and 800 nm (yellow)). The black dashed lines indicates the spectral composition of the DL emission at selected times. The inset cartoons illustrate schematically the differences in the density of scatterers between the two mediums.

To further examine the underlying mechanisms, we performed spectrally resolved measurements using a set of eight bandpass filters centered at 450, 500, …, 750, and 800 nm, each with a 40 nm bandwidth, that were placed before the detector. This bandwidth defines the spectral resolution, resulting in band-averaged DL intensities. The solid angle of collection was approximately 0.082 sr. Our automated setup allowed us to isolate and analyze the time-resolved DL signals at specific wavelengths. The limited bandwidth of the PMT, however, acts as an additional filter with non-uniform transmittance. To study relative emissions in different emission bands, one must correct for the PMT spectral response in addition to its dark count. In our case, the dark count was first subtracted from the measured signal, and the resulting intensity was then scaled by the inverse of the normalized spectral sensitivity of the PMT at each corresponding wavelength, ensuring accurate comparison across spectral bands. This procedure was implemented, and typical spectrally corrected DL emission decays for Medium 1 excited at two different wavelengths are depicted in Figure 3.

The key observation is that emissions at longer wavelengths (750 nm and 800 nm) decay much slower. This leads to a significant temporal evolution of the measured emission spectrum. In Figure 3, the black dotted lines denote the initial spectrum, that within the first milliseconds after shutting off the excitation, and the final spectral compositions of the DL emission. It is evident that the early-time spectral characteristics depend significantly on the wavelength of excitation, which should be a consequence of the radiative recombination mechanisms. As discussed in the introduction, DL emission can result from various localized and delocalized electronic transitions between energy levels. Depending on the nature of these energy levels, including shallow traps, deep traps, and deep defects, which are predominantly localized, the delay time can vary significantly across different emission wavelengths.

**Figure 4:**
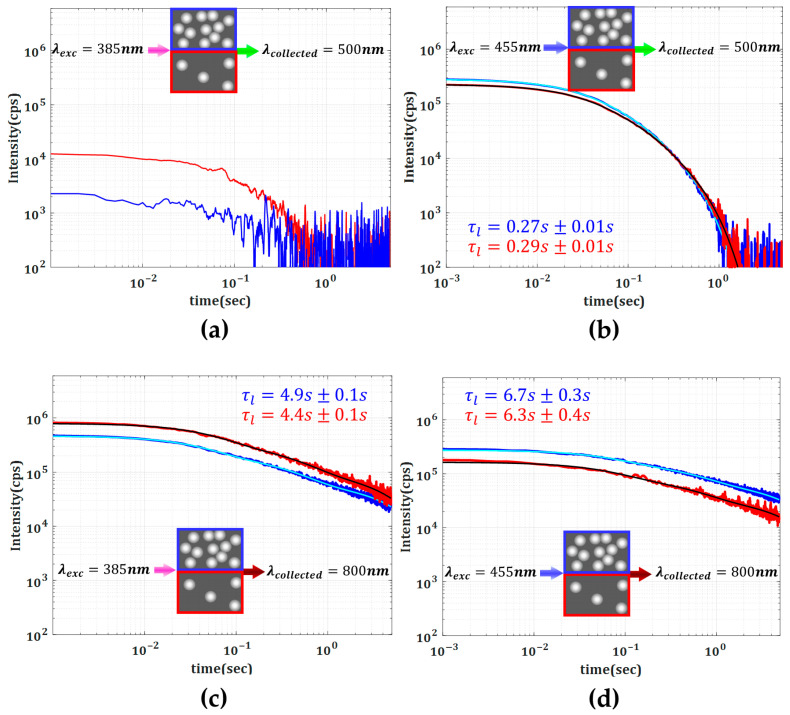
Comparison of measured DLs from Medium 1 (blue) and Medium 2 (red), excited at two different wavelengths, $\lambda_{exc}=385\;nm$ (left column, (**a**,**c**)), $\lambda_{exc}=455\;nm$ (right column, (**b**,**d**)). The emitted luminescence was collected at $\lambda_{collected}=500\;nm$ (top row, (**a**,**b**)) and $\lambda_{collected}=800\;nm$ (bottom row, (**c**,**d**)). Continuous light blue and black lines represent the corresponding multi-exponential fits. Extracted values of the longest lifetime component, $\tau_{l}$, are indicated, with uncertainties corresponding to the standard errors obtained from the fits. The inset cartoons illustrate schematically the differences in the density of scatterers between the two mediums.

Different electronic transitions result in distinct temporal behaviors at different emission wavelengths. Nevertheless, in an inhomogeneous composite such as our media, one should also expect that the intensity and temporal characteristic of DL emission are also affected by the characteristic scattering mechanisms. To better illustrate the role of scattering strength, in Figure 4, we present a direct comparison between the amplitude and the lifetime dynamics for the specific emissions at 500 nm and 800 nm corresponding to the excitation wavelengths of 385 nm and 455 nm. We also found that a quantitative analysis can be pursued by considering that the DL decays can be represented as the superposition of three independent exponentially decaying processes with a distinct rate differing by approximately one order of magnitude. To investigate the prolonged time response, we focused on the exponential term with the slowest decay rate ($\tau_{l}$).

The use of a triple-exponential model is consistent with luminescence modeling practices and effectively captures the multi-scale decay dynamics, as shown, for instance, by Kim et al. [22]. Semi-logarithmic plots of the DL intensity decay confirm the non-exponential behavior, with different decay rates dominating at different timescales. Among the various fitting strategies evaluated, the triple-exponential model consistently achieved R^2^ values exceeding 0.99, balancing accuracy and model simplicity, whereas fits with fewer or more components showed inferior performance.

## 3. Discussion of Main Observations

When examining the results in Figure 3 and Figure 4, the first observation is that the DL decays are not following a single-exponential trend, not even over narrow wavelength bands. This is an early indication of complexity due to multiple decay pathways or intricate recombination dynamics within the system [23].

A second important observation is that the longest lifetimes are associated with the lowest photon energies in both Media 1 and 2. As shown in Figure 3, beyond approximately 2 s, the only detectable emission is in the 800 nm band. In other words, the higher-energy photons decay faster while the lower-energy ones persist longer. This trend is further corroborated by data in Figure 4, where the decay rates of the DL emissions collected at 500 nm and 800 nm differ by more than an order of magnitude, highlighting the pronounced wavelength dependence of the delayed luminescence dynamics.

The excitation wavelength also plays a critical role. As previously discussed, Figure 2b clearly shows that the total emitted intensities are different when the excitation is at 385 nm or 455 nm. This difference can also be seen in Figure 3, where, for the two excitation wavelengths, the spectrally resolved DL response of Medium 1 reveals distinct differences in both the temporal and the spectral dynamics.

Another important aspect revealed in our experiments is the influence of scattering strength on the properties of DL emission. When comparing Media 1 and 2 under $\lambda_{exc}=4$55 nm, one can see that the higher TiO_2_ concentration leads to an increased emission intensity and also to an extended lifetime of low-energy photons.

**Figure 5:**
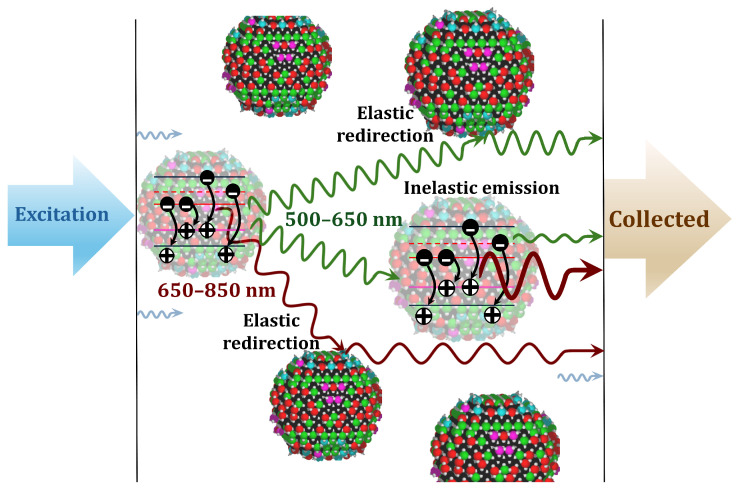
After an initial excitation during the diffusion of the pump radiation, the collected radiation is the result of successive emission, re-absorption, and re-emission processes. An increased photon path length due to multiple scattering gives rise to complex superpositions of emission, re-absorption, and re-emission events, leading to prolonged–delayed luminescence as observed in our highly scattering media.

We now provide a phenomenological interpretation of the main observations in terms of the multiple elastic and inelastic scattering processes within media exhibiting mesoscale inhomogeneities. Figure 5 presents a schematic representation of the main optical processes, including elastic scattering, absorption, emission, re-absorption, and re-emission processes.

After the initial excitation, the radiation is emitted as a result of successive elastic and inelastic processes. During their propagation through the highly inhomogeneous medium, the emitted (Stokes-shifted) photons can be re-absorbed, which increases the population of trapped electrons and holes localized at deep defect levels and, as a result, generates further Stokes-shifted photons [21,24]. This process slows down the emission of DL photons at longer wavelengths (larger Stokes shifts) [18]. Because each successive re-absorption and re-emission event introduces additional delays, the complex spectral and temporal features of DL detected from strongly scattering media depend on the distribution of photon path lengths caused by multiple elastic scattering events.

Figure 2b reveals that the luminescence decay rate can be deconvolved into multiple exponential components, which is more pronounced with the longer excitation wavelength (455 nm). This observation may be attributed to a deeper or more homogeneous excitation over the entire bulk of both scattering media at this pump wavelength, which manifests lower absorption. In other words, the distribution of excitation energy is not the same for the two wavelengths. Lower absorption permits longer photon paths through the inhomogeneous medium, i.e., the dominance of elastic processes. Therefore, excitation at 455 nm penetrates deeper and reaches a larger volume of the medium. As a result, the collected luminescence originates from the entire volume of the medium under 455 nm excitation. In contrast, when excited at 385 nm, the observed luminescence is predominantly from the input region of the medium, as the output region would be poorly excited. This broader volumetric excitation due to lower absorption at 455 nm results in enhanced DL intensity, despite the intrinsic absorption being lower compared to that at 385 nm. Consequently, the luminescence decay rate can be deconvoluted with fewer exponential components. It is worth noting that the number of emission, re-absorption, and re-emission cycles is expected to vary with the propagation distance within the medium, leading to a broader distribution of re-emission cycles for 455 nm excitation.

Figure 3 indicates a slower DL decay rate for emissions collected at longer wavelengths (750 nm and 800 nm), consistent with the expected re-absorption and re-emission processes within our scattering media. Note that larger Stokes-shifted photons must result from increased re-absorption and re-emission events, leading to longer delayed luminescence. On the other hand, slower PL decay rates at shorter emission wavelengths (450–650 nm) were observed under 455 nm excitation compared to 385 nm excitation. This observation may be attributed to the direct excitation of trapped electronic levels by 455 nm photons, which exhibit longer decay times.

For both media, excitation at 385 nm resulted in significantly lower PL intensity at 500 nm (Figure 4a,b) compared to that at 455 nm excitation. It is worth noting that for the 385 nm excitation and 500 nm emission cases (Figure 4a), the photon counts were close to the dark noise level, resulting in strong signal fluctuations. As a result, reliable lifetime values could not be extracted from the DL signal. This observation can be attributed to strong attenuation of 385 nm and 500 nm light, restricting excitation and emission to the sample’s input region. For 800 nm DL emission, a longer decay rate was observed in Medium 1 with both 385 nm and 455 nm excitations, which is attributed to stronger re-absorption and re-emission processes originating from the higher scattering strength (longer photon path length). In addition, the DL decay rate at 800 nm is significantly faster when pumped at 385 nm compared to 455 nm, possibly due to direct excitation of trapped electronic levels by the latter (455 nm). The collected PL intensity at 800 nm from Medium 2 is significantly lower when excited at 455 nm compared to 385 nm, likely due to poor absorption attenuation (higher transmission) at 455 nm excitation (see Table 1).

## 4. Conclusions

Emission of light, externally excited or spontaneous, is not commonly examined in highly scattering media where the optical properties vary significantly over spatial scales of the order of wavelength. The emission of light from such media is usually regarded as an incoherent process irrespective of their specific structural morphology. Here we have examined the re-emission of photons from complex media consisting of rutile TiO_2_ nanoparticles embedded in a polymer matrix where both elastic and inelastic scattering influence the temporal and the spectral properties of outcoming radiation.

Our results demonstrate the presence of a long-lived emission process, which is governed by the intricate interplay of carrier recombination, light transport, and energy redistribution. This DL emission is critically influenced by scattering, effective absorption, and re-absorption, which are all processes determined by the extent of typical photon path lengths within the highly scattering media. Moreover, a very strong spectral dependence leads to a characteristic temporal evolution of the DL spectral composition.

The main features of the experimental results can be interpreted in the frame of a simple model accounting for both elastic and inelastic phenomena. Increasing optical inhomogeneity leads to a slower DL decay that is attributed to successive absorption and emission of photons with large Stokes shifts. This model could be extended to other material systems and can guide the design of random media combining both phosphorescent emitters and scatterers, enabling increased absorption cross-sections and controlled delayed emission for enhanced intensity and extended lifetimes.

Finally, our demonstration constitutes an argument that simple measurements of DL spectral evolution could be convenient tools for assessing not only the chemical composition but also the structural properties of highly inhomogeneous materials.

## Figures and Tables

**Table 1:** Values of transmission coefficient (%) at different excitation wavelengths.

Transmittance (%)	385 nm	455 nm
Medium 1	0.11	3.01
Medium 2	0.47	12.46

## Data Availability

The original contributions presented in the study are included in the article, further inquiries can be directed to the corresponding author.
